# Phospholipid derived mediators and glomerulonephritis

**DOI:** 10.1155/S0962935193000134

**Published:** 1993

**Authors:** E. N. Wardle

**Affiliations:** 21 Common Road North Leigh Oxon OX8 6RD UK

## Abstract

The contributions made by the various eicosanoids, PAF, the HETES and the lipoxins to the pathophysiology of glomerulonephritis is reviewed. A case can be made for clinical trials of PAF, leukotriene and thromboxane antagonists. Combined thromboxane synthetase and thromboxane receptor antagonism would seem to be the more efficacious approach for the various disease entities.


Mediators of Inflammation, 2, 99–102 (1993)

### Phospholipid derived mediators and glomerulonephritis

E. N. Wardle

21 Common Road, North Leigh, Oxon OX8 6RD, UK

**THE contributions made by the various eicosanoids, PAF, the HETES and the lipoxins to the pathophysiology of glomerulonephritis is reviewed. A case can be made for clinical trials of PAF, leukotriene and thromboxane antagonists. Combined thromboxane synthetase and thromboxane receptor antagonism would seem to be the more efficacious approach for the various disease entities.**

**Key words:** Eicosanoids, Glomerulonephritis, 15 HETE, Leukotrienes, Lipoxins, PAF acether, Thromboxane $A_2$.

### Introduction

Glomerulonephritis is inflammation of the glomerular filtering units of the kidneys, that is caused either by deposition of immune complexes following infections or by formation of an antibody that interacts with the basement membranes of the capillaries of the glomeruli. Such inflammation will be accompanied by immediate deposition of platelet aggregates in the capillaries and a rapid infiltration with polymorphonuclear leucocytes (PMNs), and a slower infiltration of monocytes and macrophages. Thus filtration is impaired, so that the blood urea rises, and leakage of protein through permable capillaries results in a variable loss of protein in the urine called proteinuria.

We are now in a position to define the several roles of the phospholipid derived mediators, such as the various eicosanoids, PAF, the HETES and the lipoxins in glomerulonephritides. This has entailed examining their actions on the intrinsic cells of the glomeruli, like the capillary endothelial cells, the central stalk mesangial cells and the epithelial cells that lie on the urinary side of the filtration surface, which is the glomerular basement membrane (GBM). Their effects on the cells that become lodged in or that infiltrate the glomeruli, namely the platelets, PMNs and the monocyte-macrophages must also be accounted for.

As with inflammation at any site, vascular permeability that results in the leakage of protein rich fluid into the extravascular space is mediated by the combined action of vasodilator prostaglandins, that promote increased blood flow through arterioles and capillaries, and the peptidoleukotrienes that cause postcapillary venular constriction.[1] Leukotriene $D_4$ is a principal mediator,[2] and PAF[3] and the HETE molecules act synergistically, since they cause contraction of vascular endothelial cells,[4,5] and they will add to the effects of other agents like histamine, bradykinin and the anaphylatoxins that arise during the activation of complement. Loss of negatively charged heparan sulphate molecules from the walls of the glomerular capillaries is an important accessory process which means that albumin molecules are no longer subjected to 'charge repulsion' and so there is leakage of albumin. Heparitinases produced by platelets, and the lysosomal neutral proteinases of white cells that include proteoglycanases, aid such losses. At any rate involvement of platelets in the various forms of glomerulonephritis,[6] and the recruitment of PMNs followed soon by monocyte-macrophages[7] is an integral part of the process. Involvement of these phagocytic cells in free radical generating reactions is then accompanied by formation of leukotrienes,[8] as does damage to cells by complement mediated reactions.[8] Table 1 emphasizes the various roles of the phospholipid derived mediators in the complex pathophysiology of glomerulonephritides that have been deduced from animal experiments.

Survey of Table 1 reveals that the phospholipid derivatives have vascular effects that account for modulation of blood flow through the inflamed glomeruli in nephritis and that all have some effect on vascular permeability and hence proteinuria. Additionally the mediators control the responses of platelets, PMNs and infiltrating macrophages, and they alter the responses of mesangial stalk cells and thus modulate glomerular blood flow and filtration of urine. The thromboxanes even determine protein synthesis by the mesangial cells,[22] and the formation of extracellular matrix proteins like

© 1993 Rapid Communications of Oxford Ltd

**Table 1.** Pharmacological actions of phospholipid-derived mediators

| Mediator | Vascular effect | Cellular action |
|---|---|---|
| $PGI_2$ | Vasodilatation | Inhibits platelet aggregation |
| $PGE_1$ | Vasodilatation | Inhibits platelet aggregation |
| $PGE_2$ | Vasodilatation, proteinuria[9] | Causes platelet aggregation |
| $PGF_{2\alpha}$ | Vasoconstriction | Mesangial cell proliferation[10] |
| $TxA_2$ | Vasoconstriction<br>Lowers GFR and plasma flow,[11]<br>Causes proteinuria,[12]<br>Salt and water retention.[13] | Causes platelet aggregation,<br>Mesangial matrix formation,[14]<br>Enhances T-cell responses.[15] |
| $LTB_4$ | Vascular permeability[16] | Chemotaxis for white cells,[16]<br>Activation of PMNs and macrophages. |
| $LTC_4$-$D_4$ | Vascular permeability proteinuria[2] | Contraction of mesangial cells, so lowering the GFR.[17] |
| HETES | Vascular permeability[18] | 5HETE—PAF synthesis[18]<br>12HETE—chemotaxis[18]<br>15HETE—endothelial proliferation<br>15HETE—inhibition of $LTB_4$ synthesis.[17] |
| Lipoxins | $LXA_4$ vasodilator[7,20] counteracts leukotrienes.<br>$LXB_4$ lowers the GFR[20] | Reduce PMN reactivity[19] |
| PAF | Vascular permeability and proteinuria[3]<br>Loss of anionic charges.[21] | Mesangial cell contraction and reduction of the GFR[3] |

fibronectin that determine the onset of the fibrosis that leads to glomerulosclerosis and ultimate destruction.

### Specific role of the mediators in nephritides

Culture of whole glomeruli or isolated mesangial cells[23] has been used to study biochemical pharmacology. So it was shown that $PGF_{2\alpha}$[10] will act synergistically with growth factors to cause mesangial cell proliferation, and studies have been done on the interaction of cytokines and eicosanoids.[24] In the study of the various animal models of glomerulonephritis, one has to examine the effect of pharmacological mediators on physiological responses by using micropuncture studies,[25,26] and studies of the porosity of the glomerular capillary walls to dextrans of defined size and charge. In nephritides one will expect an increase in renal cortical plasma flow and yet a reduction of the glomerular filtration rate (GFR), and a marked reduction of the glomerular capillary ultrafiltration coefficient, $K_f$.[26]

In all types of nephritis there is complement activation and increased leukotriene synthesis,[1] and hence vascular permeability and a fall of GFR and an infiltration of PMNs. There is an important role for thromboxane $A_2$ in mediating the increase of renal vascular resistance during this early phase.[27,28] However soon this will be counteracted by the production of vasodilators like nitric oxide originating from endothelial cells,[29,30] increased production of $PGE_2$,[26] and the formation of lipoxin $A_4$ that causes arteriolar dilatation.[31] So then there will be increased blood flow and yet low GFR and $K_f$.

There is an early burst of glomerular synthesis of leukotrienes, following which they are suppressed.[7,8] Early production of PAF-acether contributes to the formation of leukotrienes.[32] $LTB_4$ accounts for the attraction of PMNs into the glomeruli[16] along with cytokines like interleukin-8. LTB4 accounts for attraction of monocyte-macrophages along with chemokines like monocyte chemoattractant protein, MCP-1. Leukotrienes $C_4$-$D_4$ cause deterioration of glomerular function and in the ultrafiltration coefficient,[33] $K_f$, as proven by the improvement that is achieved with a leukotriene $D_4$ antagonist.[33] Indeed it has been known for a long time that nephritis is ameliorated by rendering animals neutropenic.

There is also a free radical catalysed lipid peroxidation product 8 epi-$PGF_{2\alpha}$ (an eight isoprostane), which turns out to cause mesangial cell contraction and reduction of glomerular blood flow and filtration.[34] This mediator was discovered whilst pursuing the fact that short term cholesterol feeding to rats causes renal vasoconstriction that is overcome by a thromboxane receptor antagonist.[35]

At any rate the appearance of macrophages within the glomeruli means that there is now local production of 15HETE. 15HETE abolishes the chemotactic response of PMNs (certainly in the rat). It is converted to lipoxin $A_4$[7,36] which is antagonistic to $LTB_4$.[36,37] Hence, according to Badr,[7] the GFR is now improved. Even more significantly 15HETE synthesis is thought to account for the disappearance of PMNs from the glomeruli.[7] Its production increases progressively over days and even weeks, since there are now many local macrophages. Unfortunately it is known that their presence portends glomerulo-fibrosis.

All this means that in the later stage of nephritis poor glomerular function is more likely to be mediated by thromboxane $A_2$.[7,26] Thromboxane production also accounts for proteinuria.[36] PAF is yet another factor,[39] but we should note that many actions of PAF are probably mediated via thromboxane.[40] Selective antagonism of thromboxane will certainly improve the GFR in all the models of nephritis,[41–43] and usually proteinuria is lessened.

Much interest is now centred on the fact that thromboxanes that are released during glomerular injury lead to proliferation of mesangial cells,[41] and they promote formation of extracellular matrix proteins.[14,22,45] They also will be produced by macrophages. Concurrent lipid peroxidation leads to collagen formation.[46,47] Now it is recognized that the cytokine transforming growth factor beta leads to collagen formation and glomerulosclerosis, since TGF$\beta$ also inhibits local metalloproteinases.[48] The inter-relationships between these factors have not been explored as yet. Often mesangial cells produce type I collagen and epithelial cells produce type IV collagen.

There is ample evidence that renal perfusion is maintained during glomerulonephritis by the vasodilator action of prostaglandins,[7] albeit they also facilitate leakage of protein[27–28] through the glomeruli. It is salutary to reflect that production of prostaglandins $PGE_2$, $PGF_{2\alpha}$ and $PGI_2$ is increased as a consequence of complement mediated damage to glomerular cells,[49] as a result of local production of oxygen radicals,[50] and in response to the proinflammatory cytokines like IL-1 and TNF$\alpha$,[51] and that their ultimate role is likely to be 'cytoprotective'.

The question will arise as to whether all of these proinflammatory mediators are involved in the same way in each different histological form of nephritis. Generally this discussion applies to all forms of proliferative nephritis. In these there is infiltration of PMNs followed by monocyte-macrophages, and there is throughout an overriding role for the thromboxanes,[41–43] for thromboxane antagonists lessen the cellular proliferation and extracellular matrix deposition that leads to glomerulosclerosis and the proteinuria.

Similar mediators are certainly involved in 'membranous nephropathy' in which the capillary loop basement membranes appear thickened. This is not surprizing because involvement of the underlying epithelial cells elicits the same type of mediator response as does involvement of either endothelial or mesangial cells. A recent study has shown that PAF excretion is increased in proportion to the degree of proteinuria in membranous nephropathy in man.[52] Since it is well established that PAF excretion is increased in lupus nephritis in mice,[53] which is a proliferative cellular nephritis this again emphasizes that general statements can appertain.

### Therapy of glomerulonephritis with anti-inflammatory drugs

Owing to its ability to induce lipocortin,[54] cortisone is our most powerful anti-inflammatory and anti-allergic drug. It stops formation of the eicosanoids and of PAF and the release of cytokines like IL-1 and TNF$\alpha$. Yet effective usage is often accompanied by serious side-effects. Indeed, corticosteroids are only now used with specific intent in the nephritis of SLE (systemic lupus erythematosus) in which various lymphocyte driven auto-immune mechanisms are involved. Lymphocytes are easily suppressed by cortisone. In other forms of proliferative nephritis steroids are of little benefit.

The NSAIDs have been used to reduce proteinuria in nephrotic syndrome. Yet there is always the risk of allergic reaction and thus superimposed interstitial nephritis and acute renal failure. Hence currently the ACE inhibitors[55] are more likely to be used for reduction of proteinuria. The glomerular effects of angiotensin II are actually mediated through the thromboxanes.[56]

There is now great interest in the use of thromboxane synthetase and thromboxane receptor antagonists, for reasons that have been discussed. Increased thromboxane formation has been demonstrated in all human glomerulopathies e.g. in minimal change nephrotic syndrome, in the various proliferative nephritides and in diabetic nephropathy. In animal experiments thromboxane antagonism is successful in controlling hypertension, proteinuria and declining renal function.[41–44,57–59] The time for clinical trials has come, albeit the remark could apply also to PAF and leukotriene antagonists.

### References

1. Lianos EA. Eicosanoid biosynthesis and role in immune injury. *Prostaglandins, Leukotrienes & EFA* 1990; **41:** 1–12.
2. Badr KF, Schreiner GF, Wasserman M, Ichikawa I. Preservation of the glomerular capillary ultrafiltration coefficient during rat nephrotoxic serum nephritis by a specific leukotriene receptor antagonist. *J Clin Invest* 1988; **81:** 1701–1706.
3. Camussi G. Potential role of PAF in renal pathophysiology. *Kidney Int* 1986; **29:** 469–472.
4. Bussolino F, Camussi G, Aglietta M, *et al.* Human endothelial cells are the target for PAF induced changes in cytoskeletal structure. *J Immunol* 1987; **139:** 2439–2442.
5. Hann KV, Grossi IM, Diglio CA, Wojtukiewicz K, Taylor JD. Enhanced tumour cell adhesion to the subendothelial matrix resulting from 12-HETE induced endothelial cell retraction. *FASEB J* 1989; **3:** 2285–2287.
6. Poelstra K, Hardank MJ, Koudstaal J, Bakker WW. Intraglomerular platelet aggregation and experimental glomerulonephritis. *Kidney Int* 1990; **37:** 1500–1508.
7. Badr KF. 15-lipoxygenase products as leukotriene antagonists: therapeutic potential in glomerulonephritis. *Kidney Int* 1991; **42** (suppl 38): S101–S108.
8. Lianos A. Eicosanoids and the modulation of glomerular immune injury. *Kidney Int* 1989; **35:** 985–992.
9. Dunn MJ. The roles of angiotensin II and prostaglandins in the regulation of the glomerular filtration of albumin. *J Hypertension* 1990; **8** (suppl 1): S47–S52.

10. Mené P, Dunn MJ. Prostaglandins and rat glomerular mesangial cell proliferation. *Kidney Int* 1990; **37**: 1256–1262.
11. Wilcox CS, Welch WJ, Snellen H. Thromboxane mediates renal hemodynamic response to infused angiotensin II. *Kidney Int* 1991; **40**: 1090–1097.
12. Remuzzi G, Imberti L, Ressini M, *et al.* Increased glomerular thromboxane synthesis as cause of proteinuria in experimental nephrosis. *J Clin Invest* 1985; **75**: 94–101.
13. Pinzani M, LaffiG, Meacci E, Lavilla G, Cominelli F, Gentilini P. Intrarenal thromboxane $A_2$ generation reduces furosemide induced sodium and water diuresis in cirrhosis with ascites. *Gastroenterology* 1988; **95**: 1081–1087.
14. Bruggerman LA, Horikoshi S, Horrigan E, Ray P, Klotman PW. Thromboxane stimulates transcription of extracellular matrix proteins. *Amer J Physiol* 1991; **261**: F488–F494.
15. Ruiz P, Rey L, Spurney R, Coffman T, Viciana A. Thromboxane augmentation of allo-reactive T-cell function transplantation. 1992; **54**: 498–505.
16. Rahman MA, Nakazawa M, Emancipator SN. Increased leukotriene $B_4$ synthesis in immune injured rat glomeruli. *J Clin Invest* 1988; **81**: 1945–1952.
17. Simonson MS, Dunn MJ. Leukotriene $C_4$ and $D_4$ contract rat glomerular mesangial cells. *Kidney Int* 1986; **30**: 524–531.
18. Spector AA, Gordon JA, Moore SA. Hydroxy-eicosatetraenoic acids. *Prog Lipid Res* 1988; **27**: 271–324.
19. Brady HR, Persson U, Ballerman BJ, Brenner BM, Serhan CN. Leukotrienes stimulate neutrophil adhesion to mesangial cells: modulation with lipoxins. *Amer J Physiol* 1990; **259**: F809–F815.
20. Katoh T, Takahashi K, DeBoer DK, Serhan CN, Badr KF. Renal hemodynamic actions of lipoxins in rats. *Amer J Physiol* 1992; **263**: F436–F442.
21. Camussi G, Tetta C, Coda R, Segoloni GP, Vercellone A. Platelet activating factor induced loss of glomerular anionic charges. *Kidney Int* 1984; **25**: 73–81.
22. Mené P, Taranta A, Pugliese F, Cinotti GA, D'Agostino P. Thromboxane $A_2$ regulates protein synthesis of cultured human mesangial cells. *J Lab Clin Med* 1992; **120**: 48–56.
23. Mené P, Simonson N, Dunn MJ. Mesangial cell physiology. *Physiol Revs* 1989; **69**: 1386–1424.
24. Floege J, Topley N, Wessel K. Monokines and PDGF modulate prostanoid production in growth arrested human mesangial cells. *Kidney Int* 1990; **37**: 859–869.
25. Ichikawa T, Maddox DA, Cogan MG, Brenner BM. Dynamics of glomerular ultrafiltration in euvolaemic Munich–Wistar rats. *Renal Physiol* 1978; **i**: 121–125.
26. Badr KF. Arachidonate cyclo-oxygenase and lipoxygenase products in the mediation of glomerular immune injury. *Nephrol Dial Transp* 1991; **6**: 662–669.
27. Lianos EA, Andres G, Dunn MJ. Glomerular prostaglandin and thromboxane synthesis in rat nephrotoxic serum nephritis. *J Clin Invest* 1983; **72**: 1439–1448.
28. Stork JE, Dunn MJ. Hemodynamic roles of thromboxane $A_2$ and $PGE_2$ in glomerulonephritis. *J Pharmacol Exp Therap* 1985; **233**: 672–678.
29. Cattell V, Largen P, de Heer E, Cook T. Glomeruli synthesize nitrite in active Heymann nephritis. *Kidney Int* 1991; **40**: 847–851.
30. Cook HT, Sullivan R. Glomerular nitrite synthesis in immune complex glomerulonephritis in the rat. *Amer J Pathol* 1991; **139**: 1047–1052.
31. Garrick R, Goodman A, Shen ST, Ogunc S, Wong KY. Regulation of lipoxins and leukotriene B4 production in rat mesangial cells. *Adv Prostaglandin Thromboxane Leukotr Res* 1990; **21**: 701–706.
32. Voelkel NF, Worthen S, Henson PM, Murphy RC. Production of leukotrienes induced by platelet activating factor. *Science* 1982; **218**: 286–288.
33. Badr KF, Brenner BM, Ichikawa I. Effect of leukotrine $D_4$ on glomerular dynamics in the rat. *Amer J Physiol* 1987; **22**: F239–F243.
34. Takahashi K, Nammour TM, Fukunga M, *et al.* Glomerular actions of a free radical generated novel prostaglandin 8 epiPGF$_{2\alpha}$ in the rat. *J Clin Invest* 1992; **90**: 136–141.
35. Kaplan R, Aynedjian GS, Schlondorff D, Bank N. Renal vasoconstriction caused by short term cholesterol feeding is corrected by thromboxane antagonism or probucol. *J Clin Invest* 1990; **86**: 1707–1714.
36. Brady HR, Persson U, Ballerman BJ, Brenner BM, Serhan CN. Leukotrienes stimulate neutrophil adhesion to mesangial cells: modulation with lipoxins. *Amer J Physiol* 1990; **259**: F809–F815.
37. Badr KF, Serhan CN, Nicolau RC, Samuelsson B. The action of lipoxin $A_4$ on glomerular microcirculatory dynamics in the rat. *Biochem Biophys Res Commun* 1987; **145**: 408–414.
38. Thaiss F, Mihatsch MJ, Schoeppe W, Stahl RAK. Thromboxane mediates glomerular haemodynamics in a model of chronic glomerular disease. *Eur J Clin Invest* 1992; **22**: 182–189.
39. Lianos EA, Zanglio A. Glomerular PAF levels and origin in experimental glomerulonephritis. *Kidney Int* 1990; **37**: 736–740.
40. Badr KF, DeBaer DK, Takahashi K, *et al.* Glomerular responses to PAF in the rat: role of thromboxane $A_2$. *Amer J Physiol* 1989; **256**: F35–F43.
41. Takahashi K, Schreiner GF, Yamashita K, Christman B, Blair I, Badr KF. Predominant functional roles for thromboxane $A_2$ and prostaglandin $E_2$ during late nephrotoxic serum glomerulonephritis in the rat. *J Clin Invest* 1990; **85**: 1974–1982.
42. Cybulsky AV, Lieberthal W, Quigg RJ, Rennke HG, Salant DJ. A role for thromboxane in complement mediated glomerular injury. *Amer J Pathol* 1986; **128**: 45–51.
43. Spurney RF, Ruiz P, Klotman PE, Pisetsky DS, Coffman TM. Functional significance of thromboxane in murine lupus nephritis. *Kidney Int* 1990; **37**: 351.
44. Floege T, Topley N, Resch K. Regulation of mesangial cell proliferation. *Amer J Kidney Dis* 1991; **17**: 673–676.
45. Kohan DE. Eicosanoids: potential regulators of extracellular matrix formation in the kidney. *J Lab Clin Med* 1991; **120**: 4–6.
46. Chojkier M, Houglum K, Solis-Herruzo J, Brenner OA. Stimulation of collagen gene expression by lipid peroxidation in cultured human fibroblasts. *J Biol Chem* 1989; **264**: 16957–16962.
47. Camps J, Bargallo T, Giminez A, *et al.* Relationship between hepatic lipid peroxidation and fibrogenesis in carbon tetrachloride treated rats. *Clin Science* 1992; **83**: 695–700.
48. Roberts AB, McCune BK, Sporn MB. Transforming growth factor beta: regulation of extracellular matrix. *Kidney Int* 1992; **41**: 557–559.
49. Rother K, Hansch GM, Rauterberg EW. Complement in inflammation: induction of nephritides and progress to chromicity. *Int Arch Allergy Appl Immunol* 1991; **94**: 23–27.
50. Lianos A. Eicosanoids and the modulation of glomerular immune injury. *Kidney Int* 1989; **35**: 985–992.
51. Floege J, Topley N, Wessel K. Monokines and PDGF modulate prostanoid production in growth arrested human mesangial cells. *Kidney Int* 1990; **37**: 859–869.
52. Noris M, Benigni A, Boccardo P, Gotti E, *et al.* Urinary excretion of PAF in patients with immune mediated glomerulonephritis. *Kidney Int* 1993; **43**: 426–429.
53. Macconi D, Noris M, Benfenati E, Quaglia R, Pagliarno G, Remuzzi G. Increased urinary excretion of PAF in mice with lupus nephritis. *Life Sci* 1991; **48**: 1429–1437.
54. Flower RJ. Lipocortin: what is it and what does it mean? *Br J Rheumatol* 1992; **31**: 506–507.
55. Praga M, Hernandez E, Montyo C, Andres A, Ruilore LM, Ridicio JL. Long term beneficial effects of angiotensin converting enzyme inhibition in patients with nephrosis. *Amer J Kid Dis* 1992; **20**: 240–248.
56. Wilcox CS, Welch WJ, Snellen H. Thromboxane mediates renal haemodynamic response to infused angiotensin II. *Kidney Int* 1991; **40**: 1090–1097.
57. Spurney RF, Fan P, Ruiz P, Sanfilippo F, Pisetsky DS, Coffman TM. Thromboxane receptor blockade reduces renal injury in murine lupus nephritis. *Kidney Int* 1992; **41**: 973–982.
58. Smith SR, Creech EA, Schaffer AV. EFfects of thromboxane synthetase inhibition with CGS 13080 in human cyclosporin nephrotoxicity. *Kidney Int* 1992; **41**: 199–205.
59. De Rubertis F, Craven PA. Contribution of platelet thromboxane production to enhanced urinary excretion and glomerular production of thromboxane and to the pathogenesis of albuminuria in the streptozotocin diabetic rat. *Metabolism* 1992; **41**: 90–96.

**Received 14 January 1993; accepted in revised form 15 February 1993**
